# Is the Bite of the Heterodon, or Spreading Adder, Venomous?

**Published:** 1878-12

**Authors:** J. Schneck

**Affiliations:** Mt. Carmel, Ill.


### Clinical Reports.

### NOTES FROM PRIVATE PRACTICE.

*Is the Bite of the Heterodon, or Spreading Adder, Venomous?*

If this question were asked of a body of herpetologists, there would be but one answer; but if the medical profession were interrogated, two very positive and diametrically opposite responses would be made. So far as my observation goes, the great majority of the latter would respond in the affirmative. For example, of thirteen of the most prominent physicians of this vicinity to whom I have put this question, every one affirmed that the wound inflicted by the heterodon (or spreading adder) was venomous, and that they had never known of a contrary opinion being held. Although none of these gentlemen had treated a case of poisoning by this reptile, all had heard of cases; and yet, when the reports of such cases were thoroughly investigated, no positive connection between the supposed cause and the actual result could be demonstrated. One of the reports, on which such a belief was based, was related to me by three of the thirteen physicians referred to above, each giving the circumstances quite differently, but, of course, as he had heard them vouched for by others. All appeared equally positive that a death had been caused by the bite of a spreading adder. The details of this case, as I afterwards obtained them from the family, were these: Two boys, aged 8 and 16 years respectively, were walking, barefooted, on a prairie in which the grass was very thick and high. While thus engaged, they heard a "rattling noise" close by in the grass, which at the time very much alarmed them. After reaching home, the younger boy was suddenly taken ill, and during the next 24 hours died. A

small scratch was discovered on the left foot, which was somewhat swollen.

It so happened that on the same evening when the boys were on the prairie, and near the place where they were frightened, a spreading adder was killed.

These two facts are all the evidence there is to prove the case. The symptoms, described from memory by the parents, are those of some injury of the nervous centers, such as that of insolation, the latter being very probable, as the accident occurred upon one of the hottest days of summer.

Comment upon the history given above is quite unnecessary; yet it is on such evidence that it is generally believed throughout the country, for miles around, that Mr. D—— lost a son from the bite of a spreading adder. I might refer to other reported cases, but this is the only one I have investigated in which there was a shadow of evidence.

Of SPREADING ADDERS, Prof. E. D. Cope recognizes two species and two sub-species that occur in the United States (*Heterodon platyrinus* and the sub-species *atmodes*, and *H. simus* and the sub-species *nasicus*). Both species are ovoviviparous; all flatten the head and hiss furiously when angry. They are known by the various names of *Spreading Adder*, *Blowing Viper*, *Hog-nose Snake*, *Black Viper* and *Spotted Viper*.

It is a universally recognized fact among naturalists, so far as I have been able to learn, that none of the species are venomous. Profs. Baird and Girard, in their "Catalogue of North American Reptiles," say of them: "All the species exhibit a very threatening appearance when alive, in flattening the head, hissing violently, etc., but are perfectly harmless." Prof. Jordan says (Manual of the Vertebrates of the Northern United States): "A very variable species; when angry, it depresses and spreads the head, hissing furiously, thus exhibiting a very threatening appearance, but it is perfectly harmless." Prof. E. D. Cope, in a private letter, makes a similar remark. A thorough examination of the mouth of these serpents fails to reveal a trace of poison fangs.

In conclusion I submit the following observations:

I. In June, 1876, I put a mouse in a box with a mediumsized spotted spreading adder; the latter immediately attacked the mouse, striking him a number of times, once producing a flesh wound in the jaw; the reptile soon, however, straightened up his head, and became quite reconciled to his companion. It now was the mouse's turn, and he proceeded to revenge himself by commencing to eat up his antagonist. Beginning at the tail, he ate nearly two inches of the snake in the course of an afternoon, without encountering any further resistance. The next day the mouse was as lively as usual.

II. I put a grown cat in a box with two large black spreading adders; they immediately attacked and struck the cat several times, and then became quiet.

III. The next day I put a half grown chicken in the same box; they struck it several times, but neither chicken nor cat showed any signs of poisoning.

IV. My friend Mr. F. Stein, while putting a large spotted spreading adder from one box to another, was struck by it on the back of the hand, making a scratch nearly an inch long; the wound healed without one untoward symptom.

I should mention one of the habits of this reptile which does not seem to be generally known; it is that of feigning death. I have frequently struck them rather severely with a switch, when, after making futile efforts at attack, they would seem to bite themselves (which they really never do), and then turn on their back as if dead. A few moments of quiet are all that is necessary to revive them, when they will turn over and beat a hasty retreat.

J. SCHNECK, M. D.

MT. CARMEL, ILL.

